# From subsistence to nutritional well-being: the role of non-agricultural employment in enhancing the dietary consumption patterns of rural residents in China

**DOI:** 10.3389/fnut.2026.1793171

**Published:** 2026-03-30

**Authors:** Ruolan Yuan, Lu Wang

**Affiliations:** 1 School of Economics, Management and Law, Jiangxi Science and Technology Normal University, Nanchang, China; 2 College of Economics and Management, South China Agricultural University, Guangzhou, China

**Keywords:** food consumption, non-agricultural employment, nutritional well-being, quality upgrading, structural upgrading

## Abstract

**Introduction:**

This study investigates the impact of non-agricultural employment on food consumption upgrading among rural residents and examines the underlying mechanisms.

**Methods:**

Based on data from the 2020 China Rural Revitalization Survey (CRRS), the analysis employs a binary logit model, mediation analysis, an instrumental variable approach, and propensity score matching to empirically assess how non-agricultural employment influences improvements in food consumption structure and quality.

**Results:**

The results demonstrate that non-agricultural employment exerts a significant positive effect on both structural and qualitative aspects of rural residents’ food consumption, a finding that remains robust across multiple sensitivity tests. Mediation analysis reveals that this effect is transmitted through enhanced health awareness and improved information acquisition capacity—two key pathways linking employment to dietary improvement. Heterogeneity analysis further indicates that the magnitude of this promoting effect varies significantly across age groups and educational levels, highlighting differential responsiveness within the rural population.

**Discussion:**

These findings suggest that expanding non-agricultural employment opportunities for rural laborers, strengthening public education on nutrition and food safety, improving rural information infrastructure and market service systems, and implementing targeted guidance strategies can effectively unlock the latent potential for food consumption upgrading. Such measures are essential for advancing dietary modernization and fostering integrated rural revitalization.

## 1 Introduction

Food constitutes the foundation of human survival and development and remains a central element of residents’ consumption structure. The report of the 20th National Congress of the Communist Party of China explicitly advocates establishing a broad food perspective, which requires a precise understanding of evolving trends in population dietary patterns. This approach is not only essential for building a diversified food supply system but also serves as a key driver for advancing rural revitalization and unlocking domestic demand potential. Since the reform and opening up, food consumption among rural residents in China has undergone a historic transformation—from a state of overall scarcity to one of general abundance—and is now shifting from subsistence-oriented goals of “having enough to eat” toward quality-driven objectives of “eating well” and “eating healthily.” Specifically, direct consumption of staple grains has steadily declined, while intake of high-value animal-source and nutrient-rich foods—such as meat, poultry, eggs, aquatic products, and dairy—has continued to rise (1, 2). Nevertheless, this transition remains uneven and incomplete, with persistent disparities in food consumption between urban and rural populations. Rural dietary patterns continue to face challenges including limited dietary diversity and suboptimal nutritional balance (3, 4). According to the 2020 “Report on the Nutrition and Chronic Disease Status of Chinese Residents,” actual consumption levels of vegetables, fruits, aquatic products, eggs, and dairy among rural residents generally fall below the recommended intakes specified in the “Chinese Dietary Guidelines” (5), indicating that dietary upgrading is still in its early stages and necessitates systematic policy intervention and structural support. It should be noted that the increase in the consumption of animal-based foods does not necessarily equate to an improvement in nutritional status. Studies have shown that excessive intake of red meat and processed meats is associated with an increased risk of cardiovascular diseases and colorectal cancer (6). The current structural upgrade of this article mainly reflects the supplementation of protein intake for rural residents. However, in the long term, it is necessary to combine with dietary guidelines to advocate moderation and balance. Currently, promoting the optimization of food consumption structure and the improvement of quality among rural residents has become an important issue related to national health and rural development.

Regarding the factors influencing food consumption upgrading among residents, existing literature has provided valuable insights across multiple dimensions, including economic determinants and household characteristics. Income level is widely recognized as a key determinant (7, 8); however, its marginal effect may diminish as dietary quality improves, suggesting a potential “ceiling effect” in its ability to further enhance the dietary structure of rural residents. Other studies have employed demand system models to estimate price, income, and expenditure elasticities of food demand (9, 10). Macroeconomic factors—such as agricultural mechanization, urbanization, market development, population aging, and epidemic shocks—also play significant roles in shaping rural food consumption patterns (11). Furthermore, individual characteristics and subjective cognitive factors, including migration status, educational attainment, income expectations, and decision-makers’ nutritional attitudes—exert notable influences on the dietary composition of rural households (12, 13). With the advancement of the digital economy, digital payment capabilities have emerged as an additional factor affecting both the upgrading of food consumption patterns and nutritional intake levels in rural China (14, 15).

In the study of the socio-economic effects of non-agricultural employment, scholars have extensively examined its positive impacts on farmers’ income, poverty alleviation, agricultural investment, and overall consumption levels (16, 17). A growing body of literature has also addressed the relationship between non-agricultural employment and consumption structure (18, 19), highlighting its role in increasing the share of development- and enjoyment-oriented expenditures. Nevertheless, two critical gaps remain in the existing research. First, while much attention has been paid to the effects of non-agricultural employment on aggregate consumption or the shift from subsistence to higher-level consumption patterns, few studies have analyzed food consumption as a distinct category, particularly with regard to its differential implications for dietary structure and quality. Secondly, international nutrition transition research indicates that income growth and urbanization are often accompanied by changes in dietary structure (20). In the context of rural China, the impact of non-agricultural employment on dietary diversity has received attention (21). However, existing studies mostly focus on the connections between the production and consumption ends (22), and the attention paid to ‘quality upgrading’ behaviors such as purchasing certified food is relatively limited.

At present, rural food consumption upgrading faces multiple structural constraints. Despite sustained growth in rural per capita disposable income—providing the foundational economic basis for improved consumption—the underdeveloped commercial infrastructure, limited availability of goods and services, and high transaction costs continue to restrict rural residents’ access to diverse, high-quality, and convenient food options. In this context, the large-scale engagement of rural labor in non-agricultural employment emerges as a pivotal lens through which to understand internal rural transformation. Non-agricultural employment not only serves as a primary pathway for income enhancement but also functions as a profound experience of social mobility. By reshaping household income composition, expanding information exposure, transforming social networks, and influencing value orientations, it may exert multifaceted influences on consumption behavior that extend beyond the mere income effect (23–29). Therefore, within the dual strategic framework of rural revitalization and consumption upgrading, a systematic investigation into the mechanisms and effects of non-agricultural employment on rural residents’ food consumption upgrading holds substantial theoretical significance and practical relevance.

Building on this foundation, this paper situates its analysis within the “big food view” and the national rural revitalization strategy, drawing on data from the 2020 China Rural Revitalization Survey (CRRS) to construct a theoretical framework examining how non-agricultural employment influences food consumption upgrading. The study focuses specifically on its dual effects on dietary structural optimization and quality enhancement, while further testing the mediating roles of health awareness and information acquisition capacity. The research contributes in three key aspects: First, in terms of analytical perspective, it integrates nutrition and health considerations into the study of non-agricultural employment, thereby extending the evaluation framework from the standpoint of nutritional improvement. Second, in terms of empirical content, it not only confirms the positive impact of non-agricultural employment on both the structure and quality of food consumption but also identifies heterogeneous effects across age groups and educational attainment levels. Third, in terms of mechanism exploration, it systematically assesses the mediating pathways of enhanced health awareness and improved information acquisition, offering a deeper understanding of how non-agricultural employment contributes to dietary improvement among rural residents. These findings not only enrich the academic discourse but also provide targeted policy insights for optimizing rural dietary patterns and advancing nutritional equity.

## 2 Theoretical analysis

### 2.1 The impact of non-agricultural employment on food consumption of rural residents

With the continuous advancement of China’s rural revitalization strategy and the accelerated integration of urban and rural development, the non-agriculturalization process of rural labor force has been continuously accelerating. A large number of rural residents have deeply participated in non-agricultural economic activities through local non-agricultural employment, going out to work, or engaging in part-time work. This structural transformation not only reshapes the income sources and livelihood patterns of rural families, but also profoundly influences their consumption behaviors and lifestyles (30). Especially in the context of widespread improvement in residents’ health awareness and the increasingly diversified food supply system, rural food consumption is undergoing a transformation from “quantity satisfaction” to a focus on “structure optimization” and “quality improvement.” In this context, non-agricultural employment serves as an important channel connecting the flow of urban and rural factors, promoting farmers’ income growth and changes in concepts, and plays a crucial role in upgrading the food consumption structure and quality of rural residents.

Firstly, in terms of the upgrading of food consumption structure, non-agricultural employment significantly increases the monetization income level and income stability of rural families (31). Compared with the volatility and low return characteristics of traditional agricultural income, non-agricultural employment provides more predictable cash flow for farmers, enabling them to break away from the survival-oriented consumption mode based on basic calorie intake and shift to a more diversified and balanced dietary structure (32). Secondly, in terms of the upgrading of food consumption quality, non-agricultural employment not only enhances farmers’ willingness and ability to pay for high-value-added foods (33, 34), but also improves their sensitivity to food safety, nutritional value, and certification labels through changes in their market contact methods. Rural residents involved in the non-agricultural sector are more frequently exposed to urban consumption environments, modern retail channels, and health information dissemination networks, which promotes their gradual formation of preferences for high-quality foods such as green, organic, and non-harmful products. At the same time, the identity transformation and changes in social reference groups brought about by non-agricultural employment also strengthen their focus on the quality of life and family health, further driving them to prioritize quality attributes such as safety, traceability, and nutrition labels in food choices. Thus, the following research hypothesis is proposed:

*H1*: Non-agricultural employment contributes to the upgrading of rural residents' food consumption structure and quality.

### 2.2 The mechanism of non-agricultural employment on rural residents’ food consumption

Non-agricultural employment not only directly affects rural residents’ food consumption through income effects, but also indirectly promotes the structural and quality upgrading of food consumption through two key mediating paths: improving health awareness and enhancing information acquisition capabilities. This mechanism is rooted in the framework of human capital theory and information economics, emphasizing the significant role of non-agricultural participation in reshaping individuals’ cognitive structures and information environments. Firstly, non-agricultural employment significantly enhances rural residents’ health awareness, thereby optimizing their food consumption decisions. Compared to traditional agricultural labor, non-agricultural jobs are usually embedded in more organized and regulated socio-economic environments, where workers can develop a scientific understanding of balanced nutrition, food safety, and chronic disease prevention during their work, thus increasing their preference for high-nutrition foods and actively reducing the intake of high-salt, high-oil, and low-nutrient-density foods (35, 36). The improvement in health awareness essentially alters the food utility function of farmers, making them more inclined to consider long-term health benefits rather than short-term price costs in their consumption choices. This drives the evolution of their dietary structure towards diversity and balance, and promotes the adoption of high-quality foods such as green and organic products. Secondly, non-agricultural employment significantly enhances rural residents’ information acquisition capabilities, alleviating their information asymmetry in the food market (37). Participation in non-agricultural economic activities enables farmers to access modern information networks more frequently, including smartphone applications, social media, e-commerce platforms, and urban retail systems, thereby broadening their channels for obtaining knowledge about food nutrition, safety standards, brand certifications, and consumption trends. The improvement in information acquisition capabilities not only lowers the cognitive threshold for identifying and discriminating high-quality foods but also increases farmers’ understanding and trust in new food labels. On this basis, farmers can more effectively convert external information into consumption actions, such as purchasing high-quality fresh produce from other regions through e-commerce or prioritizing products with nutrition labels in supermarkets. Based on this, the research hypothesis is proposed:

*H2*: Non-agricultural employment promotes the upgrading of rural residents' food consumption structure and quality through enhancing health awareness and information acquisition.

## 3 Data, variable selection and model construction

### 3.1 Data sources

The data for this study is derived from the 2020 China Rural Revitalization Comprehensive Survey, which was organized and implemented by the Institute of Rural Development of the Chinese Academy of Social Sciences. This survey covers multiple aspects including agricultural production, rural development, consumption structure, and non-agricultural employment of rural households, and is highly representative. The CRRS project team randomly selected 10 sample provinces from the eastern, central, western, and northeastern regions of China, based on economic development levels and regional locations, at a ratio of 1/3 of the number of provinces in each region. These provinces include Zhejiang Province, Shandong Province, Guangdong Province, Anhui Province, Henan Province, Shaanxi Province, Guizhou Province, Sichuan Province, Ningxia Hui Autonomous Region, and Heilongjiang Province. The sample covers 50 counties (cities, districts), 150 towns, and 300 administrative villages. The project team first divided all the counties (cities, districts) in the sample provinces into 5 groups based on the per capita GDP level, and considering the even distribution in geographical space, randomly selected 1 county from each group, totaling 5 counties (cities, districts). Then, 3 towns (townships) were randomly selected from each county (city, district), and in each town (township), 1 administrative village with better economic development and 1 with poorer economic development were randomly selected. Finally, based on the household register provided by the village committee, 12 to 14 rural households were randomly selected from each administrative village using the systematic sampling method, resulting in 300 village survey questionnaires and over 3,800 rural household survey questionnaires. After data processing and the elimination of abnormal and missing observations, a total of 1,146 samples were ultimately used for analysis in this paper.

### 3.2 Variable selection

#### 3.2.1 Dependent variable

The dependent variable in this paper is the upgrading of food consumption, which is defined as the upgrading of food consumption structure and the upgrading of food consumption quality. Meat, eggs, and dairy products are important sources of high-quality protein and also important indicators of residents’ consumption upgrading. This study uses the proportion of meat, egg, and dairy consumption in total food consumption to represent the upgrading of food consumption structure. The definition of the upgrading of food consumption structure is the improvement of function and quality within the same consumption category. Therefore, the question “Has your family ever purchased certified (organic/green/harmless) food?” in the questionnaire is used to represent the quality improvement of food consumption. It should be noted that, unlike the commonly used indicators in nutrition science such as “Minimum Dietary Diversity (MDD-W)” or “Healthy Eating Index (HEI)” (38), the “structural upgrading” indicator used in this paper focuses on the economic aspect of consumption expenditure, reflecting the transformation of households from a plant-based diet to an animal-based protein consumption, rather than a strict evaluation of dietary nutritional adequacy.

#### 3.2.2 Core independent variable

Non-agricultural employment is the core independent variable in this paper. According to the relevant questions in the questionnaire, “Whether the household participates in non-agricultural employment” is selected to measure non-agricultural employment, and “The proportion of non-agricultural employment in the household” is used for robustness test.

#### 3.2.3 Mediating variable

The mediating variables in this paper are health awareness and information acquisition. The questions “Do you consciously control the intake of sugar, salt, and cooking oil?” and “Can you obtain relevant information at any time through your mobile phone or the Internet?” in the questionnaire are selected to represent health awareness and information acquisition, respectively.

#### 3.2.4 Control variables

To prevent endogeneity problems caused by omitted variables, this study comprehensively considers multiple levels of control variables. Specifically, individual characteristics include gender, marital status, health status, the educational level of the household head, and the age of the household head; family characteristics include whether the household has joined a cooperative, household income level, the proportion of the elderly, and the proportion of children; at the same time, the per capita disposable income of the village and the distance between the village and the county seat are also considered as external environmental factors. The inclusion of these control variables helps to capture the individual differences of farmers, the production and operation status of households, and the impact of the external environment on the effect of agricultural policy intervention, thereby improving the accuracy of model estimation and providing a more solid theoretical basis for policy analysis and research (see Table 1).

**Table 1:** Descriptive statistics table.

Variable	Assignment instructions	Mean	S.D.
Structural upgrade	The proportion of meat, eggs and milk consumption in total food consumption (%)	0.437	0.496
Quality upgrade	Have you ever purchased certified (organic/green/pollution-free) food? Yes = 1; No = 0	0.505	0.240
Non-farm employment	Whether to participate in non-agricultural employment, yes = 1; no = 0	0.275	0.447
Gender	Male = 1; Female = 0	0.909	0.288
Age	How old are you?	57.681	10.874
Marital status	Married = 1; Unmarried = 0	0.920	0.272
Edu	Educational attainment: 1 = Primary school or below, 2 = Junior high school, 3 = High school, 4 = College degree or above	1.837	0.781
Cooperative society	Whether to join a cooperative or not, yes = 1; no = 0	0.240	0.427
Income level	In your opinion, what level was your family’s income last year in your village? 5 = very high; 4 = relatively high; 3 = medium level; 2 = relatively low; 1 = very low	2.880	0.679
Health condition	How is your health condition? 5 = Very good; 4 = Good; 3 = Average; 2 = Poor; 1 = Very poor	3.630	0.982
Family size	Number of family members	3.165	1.455
The proportion of children	The proportion of the population under the age of 12 in the total household population	0.086	0.152
The proportion of the elderly	The proportion of the population aged 60 and above in the total household population	0.355	0.396
lnvillage_income	Per capita disposable income of villages in 2019, yuan	21,640.840	15,084.610
County_distance	The distance from the village committee to the county government, km	20.326	15.181

### 3.3 Model construction

This study focuses on the upgrading of rural residents’ food consumption, which includes the upgrading of food consumption structure and the upgrading of food consumption quality. Among them, “the upgrading of food consumption structure” is a continuous variable, so the OLS regression model is selected for empirical analysis of the upgrading of food consumption structure; “the upgrading of food consumption quality” is a binary choice variable, so the binary Logit model is more appropriate. The specific benchmark model is:


$$Y_{i}=a_{0}+\delta_{1}\text{employmen}t_{i}+\beta_{21}Z_{i}+\varepsilon_{i}$$ (1)

In Equation 1: $Y_{i}$ represents the food consumption upgrade situation of rural residents, including two variables of consumption structure upgrade and consumption quality upgrade; ${\text{employment}}_{i}$ represents the non-agricultural employment situation of rural residents; $Z_{i}$ represents other control variables that affect the explained variable.

To test the mediating role of health cognition and information acquisition in the impact of non-agricultural employment on the food consumption upgrade of rural residents, referring to the research of Baron et al. (39), a model is constructed using the stepwise regression method as follows:


$$Y_{i}=\alpha_{0}+\alpha_{1}\text{employmen}t_{i}+\alpha_{2}X_{i}+e_{i}$$ (2)

$$M_{i}=b_{0}+b_{1}\text{employmen}t_{i}+b_{2}X_{i}+\vartheta_{i}$$ (3)

$$Y_{i}=c_{0}+c_{1}\text{employmen}t_{i}+c_{2}M_{i}+c_{3}X_{i}+\mu_{i}$$ (4)

In Equations 2–4: $\text{employmen}t_{i}$ represents the core explanatory variable, namely non-agricultural employment; $M_{i}$ represents the mediating variable; $e_{i}$ , $\vartheta_{i}$ and $\mu_{i}$ are all random disturbance terms.

## 4 Empirical analysis

### 4.1 Benchmark regression

The benchmark regression results of non-agricultural employment on the upgrading of rural residents’ food consumption structure and quality are presented in Table 2. As shown in columns (1) and (3), without control variables, non-agricultural employment is significantly associated with the upgrading of rural residents’ food consumption structure and quality at the 1% statistical level. After adding control variables such as individual characteristics, household characteristics, and village characteristics, non-agricultural employment still significantly promotes the upgrading of rural residents’ food consumption structure and quality at the 1% statistical level. Therefore, non-agricultural employment is conducive to increasing the consumption of protein-rich foods such as meat, eggs, and milk among rural residents, improving their dietary nutrition; it also promotes the consumption of green, organic, and certified foods, enabling them to eat “healthier.”

**Table 2:** Benchmark regression results.

Variables	(1)	(2)	(3)	(4)
	Structural upgrade	Structural upgrade	Quality upgrade	Quality upgrade
Non-farm employment	0.274*** (0.014)	0.256*** (0.015)	0.876*** (0.086)	0.625*** (0.095)
Gender		−0.016 (0.023)		−0.148 (0.150)
Age		−0.001 (0.001)		−0.016*** (0.005)
Marital status		0.046* (0.024)		0.044 (0.166)
Edu		0.003 (0.009)		0.051 (0.057)
Cooperative society		0.019 (0.015)		0.298*** (0.094)
Income level		0.001 (0.009)		0.183*** (0.064)
Health condition		0.001 (0.007)		0.102** (0.044)
Family size		0.011*** (0.004)		0.046 (0.028)
The proportion of children		0.015 (0.043)		0.070 (0.276)
The proportion of the elderly		−0.023 (0.020)		−0.478*** (0.133)
lnvillage_income		0.000 (0.000)		0.000*** (0.000)
County_distance		−0.000 (0.000)		−0.009*** (0.003)
Constant	0.429*** (0.007)	0.405*** (0.065)	−0.401*** (0.045)	−0.387 (0.428)
Observations	1,146	1,146	1,146	1,146

Standard errors are in parentheses; *, **, and *** indicate significance at the 10%, 5%, and 1% levels, respectively.

*p < 0.1, **p < 0.05, ***p < 0.01.

Regarding the control variables, age has a negative impact on the upgrading of rural residents’ food consumption quality. This might be because the older the rural residents are, the more they are influenced by traditional consumption concepts, income constraints, insufficient information access, and habit solidification, resulting in lower awareness and willingness to pay for high-quality food, thereby inhibiting the upgrading of food consumption quality. The size of the family has a positive impact on the upgrading of rural residents’ food consumption structure. The reason is that as the family size expands, it enhances procurement efficiency, boosts income capacity, promotes the diversification of dietary needs, and optimizes the division of labor within the family. This enables rural residents to have the conditions and motivation to increase the consumption of high-nutrition foods such as high-quality proteins, fruits, and vegetables, thereby promoting the upgrading of the food consumption structure towards diversity and balance. In contrast, marital status has a significant positive impact on the upgrading of rural residents’ food consumption structure. Married rural residents, due to family responsibilities, income synergy, and enhanced health awareness, pay more attention to dietary quality and nutritional balance, thus significantly promoting the upgrading of food consumption structure towards higher quality. Additionally, cooperatives, income levels, health conditions, and village per capita income levels all have significant positive effects on the upgrading of rural residents’ food consumption quality. However, the proportion of the elderly and the distance from the county seat have significant negative effects on the upgrading of rural residents’ food consumption quality. The reasons are that cooperatives enhance supply and information accessibility, income and health conditions increase the willingness and ability to pay, while a high proportion of the elderly and distance from the county seat, due to conservative concepts and insufficient supply, restrict the upgrading. These all reflect the key influences of resources, economy, population, and location on consumption quality.

### 4.2 Endogeneity treatment

#### 4.2.1 Instrumental variable method

Due to the difficulty in controlling all the factors influencing rural residents’ food consumption, the model may have endogeneity problems caused by omitted variables. To address this, an instrumental variable for non-agricultural employment is introduced, and the instrumental variable method is adopted to overcome the potential endogeneity issues, ensuring the robustness of the regression results. The “average non-agricultural employment of other sample households within the county” is selected as the instrumental variable for non-agricultural employment. There is a neighborhood effect among households within the same county. Households exchange information about non-agricultural employment through daily communication with their neighbors, which influences their willingness to engage in non-agricultural employment and subsequently affects their non-agricultural employment choices. Therefore, the instrumental variable meets the requirement of relevance. Moreover, the non-agricultural employment experiences of other households do not directly affect the food consumption of the respondents, satisfying the requirement of exogeneity for the instrumental variable.

IV-Probit and 2SLS models are, respectively, used for regression, and the results are shown in Table 3. The IV-Probit model is used to analyze the upgrading of rural residents’ food consumption quality, while the 2SLS model is used to analyze the upgrading of rural residents’ food consumption structure. Table 3 reports the regression results of the instrumental variable method. In the models, the DWH (Durbin–Wu–Hausman) test values are all significant at the 1% statistical level, indicating that non-agricultural employment is an endogenous explanatory variable. The t-values of the instrumental variables are significant at the 1% statistical level, and the F-statistics of the first stage are all greater than the critical value of 10, indicating that the instrumental variables are correlated with the endogenous explanatory variables and there is no weak instrumental variable problem. The results of the second stage IV-Probit model show that non-agricultural employment is conducive to promoting the upgrading of rural residents’ food consumption quality. That is, after correcting for potential endogeneity bias, non-agricultural employment still has a significant positive promoting effect on the upgrading of rural residents’ food consumption quality. The results of the 2SLS two-stage least squares model show that the direction and significance level of the impact of non-agricultural employment on the upgrading of rural residents’ food consumption structure are consistent with the benchmark regression results. This indicates that after using the instrumental variable method to solve the endogeneity problem, non-agricultural employment still significantly and positively affects the upgrading of rural residents’ food consumption quality and structure. Therefore, the conclusions of this paper are robust.

**Table 3:** Regression results of instrumental variable method.

Variables	(1)	(2)	(3)
	IV-probit		2SLS
	Non-farm employment	Quality upgrade	Structural upgrade
Instrumental variable	0.793*** (0.087)	1.887*** (0.204)	0.133** (0.059)
Control variables	Yes	Yes	Yes
athrho2_1	−0.661*** (0.141)		
lnsigma2	−0.937*** (0.021)		
Constant	−1.006** (0.405)	0.475*** (0.120)	0.514*** (0.073)
Observations	1,146	1,146	1,146

Standard errors are in parentheses; *, **, and *** indicate significance at the 10%, 5%, and 1% levels, respectively.

*p < 0.1, **p < 0.05, ***p < 0.01.

#### 4.2.2 Propensity score matching method

Taking into account the robust standard errors and the number of matches of the three different matching methods comprehensively, the nearest neighbor matching method is adopted in this paper to estimate the treatment effect of non-agricultural employment on the quality upgrading of rural residents’ food consumption. The robustness of the calculation results is verified through the caliper matching method and the kernel matching method. As shown in Table 4, the ATT value before matching by the nearest neighbor matching method is 0.273, which is significant at the 1% level. After matching, the estimated results of the treatment group and the control group are 0.427 and 0.236 respectively, with a difference of 0.191. That is, compared with rural households that have not participated in non-agricultural employment, the probability of food consumption quality upgrading for rural residents who have participated in non-agricultural employment is 19.1% higher. It can be seen that non-agricultural employment helps to increase the possibility of food consumption quality upgrading for rural residents. The other two matching methods also reach the same conclusion. The effects of food consumption quality upgrading for rural residents who have participated in non-agricultural employment compared with those who have not are 17.3 and 18.9%, respectively. The results show that the model and research results used in this paper are robust.

**Table 4:** Results of propensity score matching estimation.

Matching method	Non-farm employment	Treated	Controls	ATT	S.E.	*T* value
	Before matching	0.429	0.156	0.273***	0.025	10.78
Nearest neighbor matching	After matching	0.427	0.236	0.191***	0.037	5.10
Caliper matching	After matching	0.419	0.246	0.173***	0.030	5.78
Nuclear matching	After matching	0.4268	0.237	0.189***	0.029	6.49

**Figure 1:**
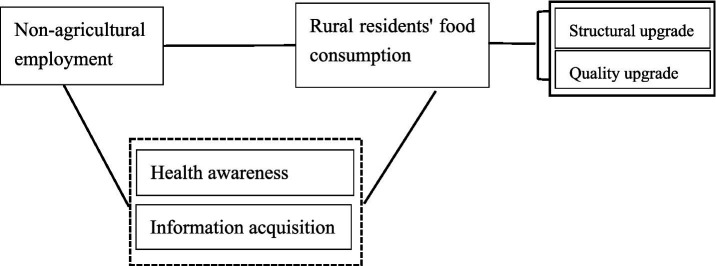
Framework diagram for theoretical analysis.

**Figure 2:**
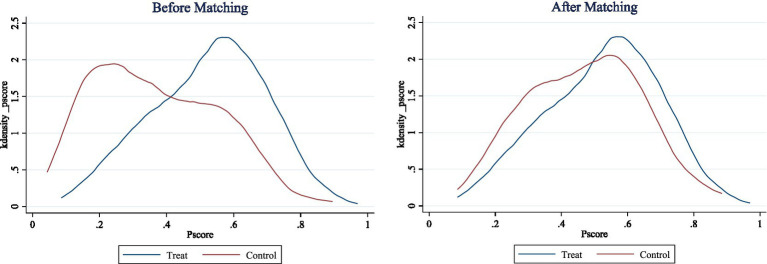
Kernel density plots before and after matching.

The principle of propensity score matching requires that the trend score must satisfy the balance. This balance includes two aspects: on the one hand, the propensity score values of the treated group farmers should be as close as possible to those of the corresponding control group farmers, and the matched samples should have good comparability, that is, they should meet the “common support” condition; on the other hand, after matching, except for whether the farmers participate in non-agricultural employment, there should be no significant differences in the mean values of other characteristic variables between the groups, that is, the “conditional independence” assumption. Figure 1 shows the kernel density plots before and after matching. After matching, the propensity scores of the treatment group and the control group overlap in a large range, and the vast majority of the observations fall within the common range. Therefore, only a small number of samples are lost during the matching process, and the matched samples have good comparability. Figure 2 shows the distribution characteristics of the absolute values of the deviations of the two types of farmers before and after matching. It can be seen that the standardized deviations of each variable have significantly decreased after matching. At the same time, this paper also conducted a balance test on the matching results and found that the deviations of most variables are less than 10%, and the t-test results of most variables show that there is no systematic difference between farmers participating in non-agricultural employment and those not participating. It can be seen that the quality of the propensity score matching in this paper is good and meets the common support assumption and the conditional independence assumption (see Table 5).

**Table 5:** Balance test results of propensity score matching method.

Variables	Treated	Control	%bias	T value	p > |t|
Gender	0.904	0.892	4.200	0.630	0.531
Age	54.238	54.814	−5.500	−0.880	0.377
Marital status	0.938	0.952	−5.200	−0.970	0.332
Edu	2.006	1.972	4.400	0.670	0.502
Cooperative Society	0.303	0.299	0.900	0.140	0.890
Income level	2.976	3.012	−5.400	−0.970	0.332
Health condition	3.769	3.757	1.200	0.210	0.831
Family size	3.195	3.096	6.800	1.080	0.280
The proportion of children	0.100	0.082	11.500	1.860	0.063
The proportion of the elderly	0.239	0.229	2.600	0.470	0.639
lnvillage_income	23,980.000	24,424.000	−3.000	−0.360	0.720
County_distance	18.081	17.506	3.900	0.730	0.467

### 4.3 Robustness test

The previous text measured the upgrading of rural residents’ food consumption quality by whether they had purchased certified (organic, green, or pollution-free) food. This paper further selects whether they produce for their own consumption as a proxy variable for the upgrading of rural residents’ food consumption quality and re-estimates. The results show (see Table 6) that non-agricultural employment has a significant promoting effect on the upgrading of rural residents’ food consumption quality, which is significantly positive at the 1% level, consistent with the conclusion of the previous text. In addition, a non-agricultural employment ratio variable is generated based on the number of non-agricultural employees in the household to replace the core explanatory variable for robustness tests. The non-agricultural employment ratio is regressed with the upgrading of food consumption structure and quality. The results show that the impact of the non-agricultural employment ratio on the upgrading of rural residents’ food consumption structure and quality remains significantly positive at the 1% statistical level. The above analysis indicates that the estimation results of this study are robust and reliable.

**Table 6:** Regression results of robustness test.

Variables	(1)	(2)	(3)
	Replace the dependent variable	Replace the independent variable	
	Quality upgrade	Structural upgrade	Quality upgrade
Non-farm employment	0.265*** (0.094)	0.255*** (0.026)	0.754*** (0.156)
Control variables	Yes	Yes	Yes
Constant	0.706* (0.403)	0.542*** (0.068)	−0.028 (0.414)
Observations	1,146	1,146	1,146

Standard errors are in parentheses; *, **, and *** indicate significance at the 10%, 5%, and 1% levels, respectively.

*p < 0.1, **p < 0.05, ***p < 0.01.

### 4.4 Mechanism of influence

The above research indicates that non-agricultural employment has a significant promoting effect on the upgrading of food consumption structure and quality for rural residents. This paper will further explore how non-agricultural employment enhances the upgrading of food consumption structure and quality for rural residents. From the perspectives of health awareness and information acquisition, this paper analyzes the specific pathways through which non-agricultural employment influences the upgrading of food consumption structure and quality for rural residents.

#### 4.4.1 Improving health awareness

The improvement of health awareness may have a significant impact on the upgrading of food consumption structure and quality for rural residents. Therefore, this paper introduces the variable of farmers’ health awareness from the perspective of cognitive improvement, and then verifies whether non-agricultural employment affects the upgrading of food consumption structure and quality for rural residents through the path of influencing health awareness. The verification results show (see Table 7) that non-agricultural employment significantly improves the health awareness of rural residents, and at the same time, non-agricultural employment can have a significant positive effect on the upgrading of food consumption structure and quality for rural residents by improving health awareness. This indicates that non-agricultural employment not only promotes consumption upgrading through economic channels such as increasing income, but also reshapes farmers’ dietary preferences and consumption decisions through non-economic paths such as expanding information contact and improving health concepts. Health awareness plays an important mediating role in this process, highlighting the key significance of “cognitive empowerment” in promoting the modernization transformation of rural residents’ diets.

**Table 7:** Regression results of mediating effect test based on health cognition.

Variables	(1)	(2)	(3)
	Health cognition	Structural upgrade	Quality upgrade
Non-farm employment	0.644***	0.239*** (0.015)	1.045*** (0.112)
	(0.117)		
Health cognition		0.100*** (0.014)	0.503*** (0.098)
Control variables	Yes	Yes	Yes
Constant	0.116 (0.438)	0.388*** (0.063)	−0.863** (0.439)
Observations	1,146	1,146	1,146

Standard errors are in parentheses; *, **, and *** indicate significance at the 10%, 5%, and 1% levels, respectively.

*p < 0.1, **p < 0.05, ***p < 0.01.

#### 4.4.2 Strengthening information acquisition

This paper analyzes from the perspective of enhancing information acquisition how non-agricultural employment influences the upgrading of food consumption structure and quality among rural residents. The results show (see Table 8) that non-agricultural employment has a significant positive impact on farmers’ information acquisition, and information acquisition plays a mediating role in the effect of non-agricultural employment on the upgrading of food consumption structure and quality among rural residents. This might be because non-agricultural employment enables rural residents to have more contact with urban markets, modern media, and diverse social networks, broadening their channels for obtaining information on food nutrition, safety standards, and consumption trends; and the enhancement of information acquisition ability helps farmers more accurately identify the value of high-quality food, update their consumption concepts, and optimize their dietary choices, thereby promoting the transformation of food consumption from “quantity” to “quality” and “rational structure.” Therefore, information acquisition plays a crucial mediating role in the process by which non-agricultural employment affects the upgrading of rural residents’ food consumption.

**Table 8:** Regression results of mediating effect test based on information acquisition.

Variables	(1)	(2)	(3)
	Information acquisition	Structural upgrade	Quality upgrade
Non-farm employment	0.139** (0.055)	0.249*** (0.015)	0.591*** (0.097)
Information acquisition		0.039*** (0.008)	0.384*** (0.053)
Control variables	Yes	Yes	Yes
Constant	2.311*** (0.233)	0.352*** (0.066)	−1.147*** (0.443)
Observations	1,146	1,146	1,146

Standard errors are in parentheses; *, **, and *** indicate significance at the 10%, 5%, and 1% levels, respectively.

*p < 0.1, **p < 0.05, ***p < 0.01.

### 4.5 Heterogeneity analysis

Further consideration is given to the impact of non-agricultural employment on the upgrading of food consumption structure and quality among rural residents, taking into account the heterogeneity of intergenerational groups and educational attainment.

#### 4.5.1 Analysis considering intergenerational heterogeneity

With the transformation of the rural social economy in China, the intergenerational differentiation of rural non-agricultural employment labor force is obvious, and different generational rural households show significant differences in terms of values, emotional preferences, and behavioral logic. Therefore, it is necessary to examine the differences in the impact of non-agricultural employment on the upgrading of food consumption structure and quality among rural residents of different generations. This paper divides rural residents into the youth group (under 45 years old), the middle-aged group (45–59 years old), and the elderly group (over 60 years old) based on their age, and conducts separate sample regressions. The results are shown in Table 9. Non-agricultural employment has a significant positive effect on the upgrading of food consumption structure for rural residents in the youth group, middle-aged group, and elderly group. At the same time, it significantly promotes the upgrading of food consumption quality for rural residents in the middle-aged group and elderly group, but has no impact on the upgrading of food consumption quality for rural residents in the youth group. This is mainly because different age groups have differences in consumption goals, income stages, health concerns, and information sensitivity. Although the youth group has a high proportion of non-agricultural employment and strong information acquisition capabilities, some young people are still in the early stage of income accumulation and are relatively cautious about paying for high-premium quality food. At the same time, the consumption of the youth group is more inclined towards convenience, diversity and social attributes. On the other hand, young workers often choose to eat out or rely on external catering services more frequently, making it difficult for household-level certified food purchase data to truly reflect their actual dietary intake, thus resulting in a contradiction between household consumption data and intuition. On the other hand, middle-aged groups have heavy family responsibilities and strong health awareness. Under the dual improvement of income and information brought by non-agricultural employment, they are more inclined to choose safe and nutritious high-quality food for their families. Although the elderly group has relatively conservative consumption concepts, after non-agricultural employment significantly improves their economic conditions and health awareness, they also pay more attention to the safety and nutritional value of food, thereby promoting quality upgrades. Therefore, non-agricultural employment has a universal promoting effect on the upgrading of food consumption structure, but the impact on quality upgrades is not significant in the youth group, reflecting age heterogeneity.

**Table 9:** Heterogeneity analysis of intergenerational groups.

Variables	Youth group		Middle-aged group		Senior group	
	(1)	(2)	(3)	(4)	(5)	(6)
	Structural upgrade	Quality upgrade	Structural upgrade	Quality upgrade	Structural upgrade	Quality upgrade
Non-farm employment	0.213*** (0.053)	0.482 (0.354)	0.278*** (0.021)	0.620*** (0.138)	0.238*** (0.023)	0.643*** (0.146)
Control variables	Yes	Yes	Yes	Yes	Yes	Yes
Constant	0.817*** (0.216)	−0.529 (1.555)	0.449*** (0.092)	0.159 (0.633)	0.356*** (0.099)	−0.802 (0.635)
Observations	133	133	535	535	478	478

Standard errors are in parentheses; *, **, and *** indicate significance at the 10%, 5%, and 1% levels, respectively.

*p < 0.1, **p < 0.05, ***p < 0.01.

#### 4.5.2 Analysis considering the heterogeneity of educational attainment

Theoretically, rural residents with different educational levels have differences in cognitive and behavioral abilities, and thus vary in their use of digital technology to obtain information and improve the structure and quality of their food consumption. This study divides the educational level of the household head into two groups: low educational attainment (below junior high school) and high educational attainment (junior high school and above), and conducts regressions for each group. The results are shown in Table 10. Non-agricultural employment significantly promotes the upgrading of the food consumption structure and quality for both low and high educational attainment rural residents. However, the effect on the quality upgrade of food consumption for low educational attainment rural residents is greater, while the effect on the structure upgrade of food consumption for high educational attainment rural residents is more pronounced. The reason is that low educational attainment households originally have weaker information acquisition capabilities and relatively lagging health and quality awareness. Non-agricultural employment significantly broadens their channels to access external markets and modern consumption concepts, effectively compensating for the cognitive deficiencies caused by insufficient education, thereby generating a stronger marginal effect on their food quality upgrade (such as choosing green, safe, and certified foods). In contrast, high educational attainment households already possess strong information processing capabilities and a solid foundation in nutrition knowledge. Non-agricultural employment mainly enhances their income levels and consumption capabilities, further optimizing dietary diversity and balance, and thus has a more prominent promoting effect on the structure upgrade of food consumption (such as increasing the consumption of high-quality categories like meat, dairy, and fruits). This reflects that non-agricultural employment differentially promotes food consumption upgrading among different educational groups through complementary mechanisms (compensating for weaknesses vs. strengthening advantages).

**Table 10:** Heterogeneity analysis of educational attainment.

Variables	Low educational attainment		High level of education	
	(1)	(2)	(3)	(4)
	Structural upgrade	Quality upgrade	Structural upgrade	Quality upgrade
Non-farm employment	0.247*** (0.017)	0.652*** (0.105)	0.292*** (0.036)	0.468** (0.232)
Control variables	Yes	Yes	Yes	Yes
Constant	0.448*** (0.070)	−0.400 (0.459)	0.392** (0.154)	0.449 (1.058)
Observations	950	950	196	196
R-squared	0.259		0.349	

Standard errors are in parentheses; *, **, and *** indicate significance at the 10%, 5%, and 1% levels, respectively.

*p < 0.1, **p < 0.05, ***p < 0.01.

### 4.6 Extended analysis

To further identify in which specific food categories the impact of non-agricultural employment on the upgrading of rural residents’ food consumption structure lies, this study further subdivided the structural upgrading variable into specific food categories (red meat, poultry meat, aquatic products, eggs, and dairy products). The regression results are shown in Table 11. The impact of non-agricultural employment on various food consumption of rural residents shows significant heterogeneity. Specifically, non-agricultural employment significantly increased the consumption proportion of red meat and dairy products among rural residents, indicating that farmers involved in non-agricultural employment tend to increase the intake of high-protein and high-nutrition-density animal-based foods, reflecting their stronger payment capacity and consumption preferences in dietary structure optimization. However, the impact of non-agricultural employment on poultry meat, aquatic products, and eggs was not significant, suggesting that there was no obvious promoting effect in these categories, possibly related to price sensitivity, consumption habits, or market accessibility. Overall, non-agricultural employment mainly achieves the upgrading of food consumption structure by promoting the growth of red meat and dairy product consumption, demonstrating its preference guiding role in specific high-protein food, and further revealing the differentiated path of non-agricultural employment influencing the dietary structure of rural residents.

**Table 11:** The impact of non-agricultural employment on the consumption of various food items by rural residents.

Variables	Red meat	Poultry	Aquatic products	Eggs	Dairy
Non-farm employment	0.436*** (0.159)	−0.100 (0.092)	−0.062 (0.086)	0.027 (0.096)	0.327* (0.169)
Control variables	Yes	Yes	Yes	Yes	Yes
Constant	1.981*** (0.689)	1.367*** (0.396)	0.449 (0.371)	1.686*** (0.415)	0.667 (0.731)
Observations	1,140	1,140	1,140	1,140	1,140
R-squared	0.009	0.021	0.014	0.057	0.012

Standard errors are in parentheses; *, **, and *** indicate significance at the 10%, 5%, and 1% levels, respectively.

*p < 0.1, **p < 0.05, ***p < 0.01.

## 5 Conclusions and suggestions

With the accelerating trend of food consumption upgrading among rural residents, non-agricultural employment has become a key factor in promoting the optimization of their dietary structure and the improvement of consumption quality. This study reveals the impact of non-agricultural employment on the upgrading of rural residents’ food consumption structure and quality through empirical analysis. The findings show that non-agricultural employment significantly promotes the upgrading of rural residents’ food consumption structure and quality at the 1% significance level. After conducting robustness tests using instrumental variable methods, propensity score matching methods, and by replacing the dependent and independent variables, non-agricultural employment still significantly promotes the upgrading of rural residents’ food consumption structure and quality. The mediation mechanism test indicates that non-agricultural employment further positively affects the upgrading of rural residents’ food consumption structure and quality by improving health awareness and enhancing information acquisition capabilities. The heterogeneity analysis shows that non-agricultural employment has varying degrees of promoting effects on the upgrading of food consumption structure and quality among rural residents of different age groups and educational levels. Specifically, it has no impact on the food consumption quality upgrading of young rural residents, while it has a greater effect on the food quality upgrading of low-educated rural residents and a more significant impact on the food consumption structure upgrading of high-educated rural residents.

Based on the above research results, the following policy recommendations are proposed to effectively promote the upgrading of rural residents’ food consumption: First, expand non-agricultural employment channels by developing county economies, rural industries, and vocational skills training to increase farmers’ income levels and lay a solid foundation for consumption capacity. Second, strengthen health nutrition and food safety education and publicity, especially targeting the elderly and low-education groups, and use village platforms and new media to popularize dietary knowledge and enhance health awareness. Third, improve rural information and logistics infrastructure, promote the coverage of broadband and cold chain logistics, support the expansion of high-quality food supply channels such as e-commerce and chain supermarkets to rural areas, and lower the threshold for accessing high-quality food. Fourth, implement differentiated guidance strategies, promote convenient and healthy food to the youth, strengthen the publicity of the connection between food safety and chronic disease prevention for the middle-aged and elderly, focus on demonstration and drive for low-education farmers, and provide refined consumption information for high-education farmers. Through a coordinated path of “employment for income increase, cognition for transformation, information for accessibility, and policy for precision,” systematically promote the upgrading of rural food consumption from “quantity” to “quality” and “structural optimization,” and facilitate the deep integration of Healthy China and rural revitalization.

## Data Availability

The original contributions presented in the study are included in the article/supplementary material, further inquiries can be directed to the corresponding author.
